# Enhanced myocardial blood flow in ischemic cardiomyopathy by a slow-release synthetic prostacyclin agonist combined with coronary artery bypass grafting: The first human study in a Phase I/IIa clinical trial

**DOI:** 10.3389/fcvm.2023.1047666

**Published:** 2023-01-25

**Authors:** Takuji Kawamura, Daisuke Yoshioka, Masashi Kawamura, Ryohei Matsuura, Ai Kawamura, Yusuke Misumi, Tsubasa Mikami, Yoshiki Sawa, Shigeru Miyagawa

**Affiliations:** Department of Cardiovascular Surgery, Osaka University Graduate School of Medicine, Suita, Japan

**Keywords:** ischemic cardiomyopathy, coronary artery bypass grafting, myocardial blood flow, functional recovery, first in human (FIH) trials

## Abstract

**Background:**

YS-1402, which is a polymerized form of the synthetic prostacyclin agonist ONO-1301, has been proven in several preclinical studies to induce therapeutic effects for patients with ischemic cardiomyopathy (ICM). In this human study, we assessed the safety, tolerability, and efficacy of YS-1402, combined with coronary artery bypass grafting (CABG), for ICM.

**Methods:**

Twenty-four patients with ICM whose left ventricular ejection fraction was <40% with an indication for CABG were double-blindly assigned to four groups: placebo, 10-mg YS-1402, 30-mg YS-1402, and 100-mg YS-1402. YS-1402 or placebo medications were administered on the surface of the left ventricle at the time of the CABG. Pre- and postoperative cardiac function and myocardial blood flow were assessed for 6 months postoperatively, along with a safety assessment.

**Results:**

No severe adverse events were related to YS-1402. The maximum blood concentration of ONO-1301 was less than that of the no observable adverse effect level. Significantly increased myocardial blood flow (MBF) and cardiac function were observed in the YS-1402 group 26 weeks postoperatively, although no improvement in MBF occurred in the placebo group.

**Conclusion:**

This Phase I/IIa parallel group-controlled, dose-escalation study of YS-1402 combined with CABG for ICM demonstrated the safety, tolerability, and potential efficacy of YS-1402.

## Introduction

Heart failure due to ischemic cardiomyopathy (ICM) has a poor prognosis, despite advances in medicine (1). Coronary artery bypass grafting (CABG) is an established treatment strategy for ICM to induce cardiac functional recovery, which improves prognosis by increasing myocardial blood flow (2, 3). Nevertheless, some patients with ICM treated with CABG have a poor prognosis because of the limited recovery of cardiac function (4). In these patients, an impaired peripheral circulatory network in the myocardium due to coronary atherosclerosis could limit the rescue of the hibernating myocardium. For patients refractory to coronary revascularization, regenerating a new myocardial blood flow network could improve cardiac function and consequently achieve a better prognosis.

YS-1402 is a poly-lactic-co-glycolic acid that is a polymerized form of ONO-301, designed to achieve a sustained-release system (5). ONO-1301 is a synthetic prostacyclin agonist consisting of structures with thromboxane A2 inhibitory activity (5). It was initially developed as an oral antiplatelet drug; however, Phase I of its clinical trial failed because of a high incidence of diarrhea and hypotension. YS-1402 administered directly to the heart as a sustained-release drug induces the release of multiple protective cytokines such as vascular endothelial growth factor (VEGF), hepatocyte growth factor, and stromal cell-derived factor 1. These cytokines contribute to the reconstruction of the microvascular network and the consequent improvement of blood flow in pigs with experimentally induced ischemic myocardium, thereby demonstrating therapeutic effects for ICM in several preclinical studies (6–8).

In this study, we hypothesized that YS-1402 combined with CABG would improve the myocardial blood flow of patients with ICM to induce functional recovery. Therefore, the primary objective of the study was to assess the safety of adverse events possibly caused by this administration. Subsequently, because the previous Phase I clinical trial of the oral intake of ONO-1301, which is the active compound of YS-1402, revealed the maximum plasma concentration that caused adverse effects, the secondary objective was to confirm the pharmacokinetics of ONO-1301 through the administration of YS-1402. Finally, the assessment of the therapeutic efficacy of YS-1402 for ICM was planned as an exploratory objective. To the best of our knowledge, we believe this study is the first human study in the Phase I/IIa clinical trial, entitled “Parallel group controlled, dose-escalation study to assess the safety, tolerability, and efficacy of YS-1402 combined with CABG for ischemic cardiomyopathy.”

## Results

In this clinical trial, 24 patients were enrolled, after screening using the inclusion/exclusion criteria. They were subsequently assigned to four groups, based on the YS-1402 dose, as follows: the placebo, 10-mg YS-1402, 30-mg YS-1402, and 100-mg YS-1402 groups. All patients were assigned in a double-blinded manner. Of these 24 patients, one patient in the 100-mg group was withdrawn from the trial 8 weeks postoperatively because of undergoing a surgical treatment that was judged to affect the results of this clinical trial. Another patient in the placebo group stopped visiting the hospital and canceled participation in the trial at 26 weeks postoperatively. The other 22 patients completed the scheduled trial period.

### Patients’ characteristics

The preoperative patients’ characteristics are shown in Table 1. The median age of patients in the placebo and YS-1402 group was 69 and 64 years, respectively. The frequency of coronary risk factors such as smoking, diabetes mellitus, hypertension, hyperlipidemia, and CKD was not significantly different between the groups (Table 1).

**TABLE 1:** Patient characteristics.

				YS-1402			
	**Placebo**	**YS-1402**	***P*** -value*	**10 mg**	**30 mg**	**100 mg**	***P*** -value**
n	6	18	–	6	6	6	–
Age, years	69 (62–74)	64 (57–73)	0.5	62 (52–70)	65 (58–77)	67 (57–73)	0.78
Male, n (%)	5 (83%)	17 (94%)	0.42	5 (83%)	6 (100%)	6 (100%)	0.4
BMI, kg/m^2^	23 (19–25)	24 (19–27)	0.74	21 (19–24)	23 (19–27)	26 (23–28)	0.31
Smoking, n (%)	3 (50%)	16 (89%)	0.05	5 (83%)	6 (100%)	5 (83%)	0.19
DM, n (%)	4 (67%)	10 (56%)	0.63	1 (17%)	4 (67%)	5 (83%)	0.09
HT, n (%)	3 (50%)	7 (39%)	0.63	1 (17%)	2 (33%)	4 (67%)	0.31
HL, n (%)	6 (100%)	16 (89%)	0.27	6 (100%)	4 (67%)	6 (100%)	0.11
CKD, n (%)	1 (17%)	2 (11%)	0.73	1 (17%)	1 (17%)	0 (0%)	0.6

Values are n, median (interquartile range), or n (%). *Placebo vs. YS-1402 as a whole, **between four groups; placebo, 100 mg, 30 mg, 10 mg of YS-1402. BMI, body mass index; DM, diabetes mellitus; HT, hypertension; HL, hyperlipidemia; CKD, chronic kidney disease.

### Operative procedures

The detailed CABG procedure is shown in Table 2. The off-pump procedure was performed for 7 of 24 patients, and intra-aortic balloon pump support was performed for 4 of 24 patients with no statistical significance between the placebo and YS-1402 groups or between the four groups. The number of anastomoses, target vessels of the coronary artery, and choice of graft were not significantly different between the placebo and YS-1402 groups or between the four groups (Table 2). With regard to the site on which the YS-1402 sheets were placed, the basal anterior wall (number 1) and the septal wall (numbers 2, 3, 8, 9, and 14) was not available because the approach was from surface of the LV. The YS-1402 sheets were placed according to the ischemic site of the LV without significant differences between the placebo and YS-1402 groups or between the four groups (Table 3).

**TABLE 2:** Operative procedure.

					**YS-1402**			
		**Placebo**	**YS-1402**	***P*** -value*	**10 mg**	**30 mg**	**100 mg**	***P*** -value**
n		6	18	–	6	6	6	–
Off pump, n (%)		2 (33%)	5 (28%)	0.8	4 (67%)	1 (17%)	0 (0%)	0.07
IABP, n (%)		1 (17%)	3 (17%)	1	2 (33%)	0 (0%)	1 (17%)	0.49
Anastomosis, n		3.5 (2–4)	3 (2.8–4)	0.91	3 (2.75–4.25)	4 (2.5–4)	3 (2–3.5)	0.94
Target	LAD, n (%)	5 (83%)	17 (94%)	0.42	5 (83%)	6 (100%)	6 (100%)	0.53
	LCx, n (%)	6 (100%)	15 (83%)	0.17	6 (100%)	4 (67%)	5 (83%)	0.24
	RCA, n (%)	5 (83%)	14 (78%)	0.77	5 (83%)	4 (67%)	5 (83%)	0.86
Graft	ITA, n (%)	6 (100%)	17 (94%)	0.44	6 (100%)	6 (100%)	5 (83%)	0.37
	RA, n (%)	3 (50%)	11 (61%)	0.63	6 (100%)	2 (33%)	3 (50%)	0.1
	SVG, n (%)	3 (50%)	7 (39%)	0.63	1 (17%)	3 (50%)	3 (50%)	0.52

Values are n, median (interquartile range), or n (%). *Placebo vs. YS-1402 as a whole, **between four groups; placebo, 100 mg, 30 mg, 10 mg of YS-1402. IABP, intra-aortic balloon pumping; LAD, left anterior descending artery; LCx, left circumflex artery; RCA, right coronary artery; ITA, internal thoracic artery; RA, radial artery; SVG, saphenous vein graft.

**TABLE 3:** YS-1402 site.

					**YS-1402**			
		**Placebo**	**YS-1402**	***P*** -value*	**10 mg**	**30 mg**	**100 mg**	***P*** -value**
17-segment model	#1	0 (0%)	0 (0%)	–	0 (0%)	0 (0%)	0 (0%)	–
	#2	0 (0%)	0 (0%)	–	0 (0%)	0 (0%)	0 (0%)	–
	#3	0 (0%)	0 (0%)	–	0 (0%)	0 (0%)	0 (0%)	–
	#4	3 (50%)	7 (39%)	0.63	4 (67%)	3 (50%)	0 (0%)	0.10
	#5	3 (50%)	6 (33%)	0.47	4 (67%)	1 (17%)	1 (17%)	0.19
	#6	1 (17%)	0 (0%)	0.09	0 (0%)	0 (0%)	0 (0%)	0.41
	#7	2 (33%)	9 (50%)	0.47	2 (33%)	3 (50%)	4 (67%)	0.60
	#8	0 (0%)	0 (0%)	–	0 (0%)	0 (0%)	0 (0%)	–
	#9	0 (0%)	0 (0%)	–	0 (0%)	0 (0%)	0 (0%)	–
	#10	3 (50%)	7 (39%)	0.63	4 (67%)	3 (50%)	0 (0%)	0.10
	#11	3 (50%)	6 (33%)	0.47	4 (67%)	1 (17%)	1 (17%)	0.18
	#12	3 (50%)	9 (50%)	1.00	2 (33%)	3 (50%)	4 (67%)	0.72
	#13	3 (50%)	9 (50%)	1.00	2 (33%)	3 (50%)	4 (67%)	0.71
	#14	0 (0%)	0 (0%)	–	0 (0%)	0 (0%)	0 (0%)	–
	#15	3 (50%)	9 (50%)	1.00	4 (67%)	3 (50%)	2 (33%)	0.71
	#16	5 (83%)	11 (61%)	0.31	2 (33%)	3 (50%)	6 (100%)	0.06
	#17	3 (50%)	10 (56%)	0.81	2 (33%)	3 (50%)	5 (83%)	0.36

Values are n, median (interquartile range), or n (%). *Placebo vs. YS-1402 as a whole, **between four groups; placebo, 100 mg, 30 mg, 10 mg of YS-1402.

### No significant severe adverse events occurred in the YS-1402 groups

During the 26-week postoperative observation period, no patient died. Two cases of cardiac tamponade, one case of mediastinitis, one case of wound infection, four cases of pneumonia, and one case of acute kidney failure requiring hemodialysis occurred in the perioperative phase (Table 4). One case of cerebral hemorrhage in the placebo group occurred 24 days postoperatively. One case of cholangitis in the placebo group occurred 42 days postoperatively. Thus, no significant differences in any adverse events categorized as severe occurred between the placebo and YS-1402 groups or between the four groups. Perioperative serum chemistry tests such as aspartate transaminase (AST), alanine transaminase (ALT), and Creatine Kinase showed no significant differences between the placebo and YS-1402 groups or between the four groups (Supplementary Figure 4).

**TABLE 4:** Adverse events.

					YS-1402			
		**Placebo**	**YS-1402**	***P*** -value*	**10 mg**	**30 mg**	**100 mg**	***P*** -value**
Severe adverse events	n	6	18	–	6	6	6	–
	Death, n (%)	0 (0%)	0 (0%)	–	0 (0%)	0 (0%)	0 (0%)	–
	Cardiac tamponade, n (%)	0 (0%)	2 (11%)	0.27	0 (0%)	0 (0%)	2 (34%)	0.09
	Mediastinitis, n (%)	0 (0%)	1 (6%)	0.44	0 (0%)	1 (17%)	0 (0%)	0.37
	Wound infection, n (%)	0 (0%)	1 (6%)	0.44	0 (0%)	0 (0%)	1 (17%)	0.37
	Pneumonia, n (%)	1 (17%)	3 (17%)	1.00	2 (33%)	1 (17%)	0 (0%)	0.49
	Cerebral hemorrhage, n (%)	1 (17%)	0 (0%)	0.09	0 (0%)	0 (0%)	0 (0%)	0.37
	Cholangitis, n (%)	1 (17%)	0 (0%)	0.09	0 (0%)	0 (0%)	0 (0%)	0.37
	Acute kidney failure, n (%)	0 (0%)	1 (6%)	0.44	0 (0%)	0 (0%)	1 (17%)	0.37
	Pleural effusion, n (%)	2 (33%)	16 (89%)	0.01	4 (67%)	6 (100%)	6 (100%)	0.02
	Diarrhea, n (%)	2 (33%)	2 (11%)	0.23	0 (0%)	2 (33%)	0 (0%)	0.19

Values are n (%).

*Placebo vs. YS-1402 as a whole, **between four groups; placebo, 100 mg, 30 mg, 10 mg of YS-1402.

Nonetheless, 18 cases of pleural effusion requiring diuretics or drainage were observed at the perioperative phase with significant differences between the placebo and the YS-1402 groups (*p* = 0.01) and between the four groups (*p* = 0.02) (Table 4). Diarrhea, which occurred in the previous Phase I clinical trial of oral ONO-1301 intake, was observed in four patients without a significant difference between the groups (Table 4).

### Blood concentration of ONO-1301 was below the safety margin in the YS-1402 groups

**FIGURE 1:**
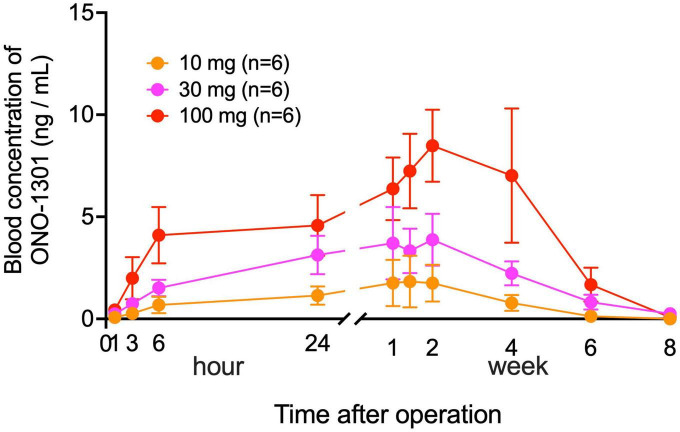
Serial changes in blood concentration of ONO-1301. Serial changes postoperatively in the blood concentration of ONO-1301 in each group (10-mg YS-1402, 30-mg YS-1402, and 100-mg YS-1402). The data are presented as the mean ± the standard error.

Serial changes in blood concentration of ONO-1301 were measured in the YS-1402 groups for 8 weeks postoperatively. The concentration of ONO-1301 was detectable from 1 h until 6 weeks postoperatively in a dose-dependent manner (Figure 1). In each group, the peak concentration of ONO-1301 was observed at 2 weeks after administration, which confirmed that YS-1402 on the heart could act as a drug by slowly releasing ONO-1301 in clinical situations. Moreover, the maximum concentration (C_max_) of ONO-1301 in this study was lower than the no observable adverse effect level (NOAEL) in the oral administration Phase I study.

### Improved LVEF after YS-1402 administration combined with CABG for ICM

**FIGURE 2:**
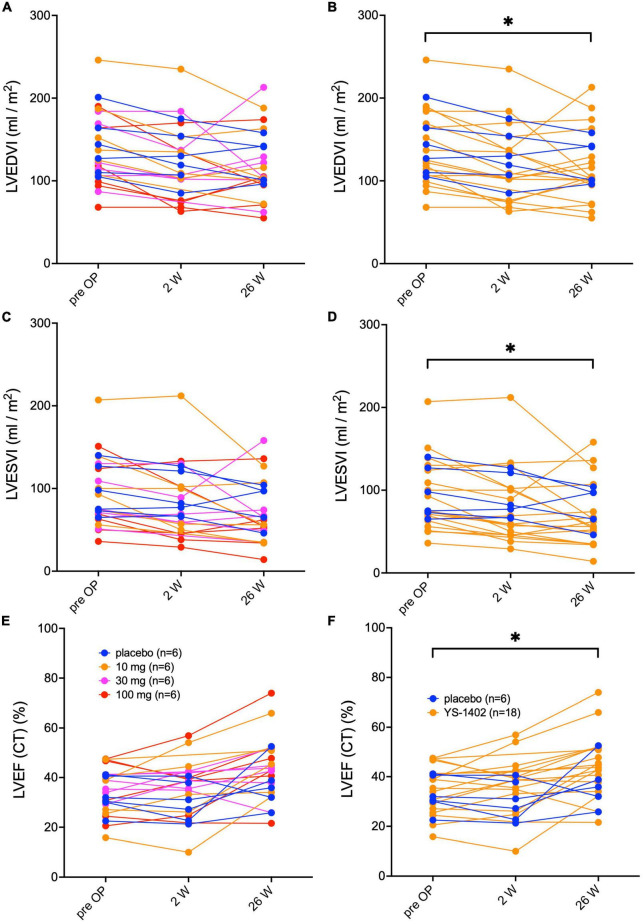
Serial assessments of left ventricular volume and ejection fraction. Serial changes in the left ventricular end-diastolic volume index (LEVI) in each of the four groups **(A)** and in the combined YS-1402/placebo group **(B)**, the ventricular end-systolic volume index (LVESVI) in each of the four groups **(C)** and in the combined YS-1402/placebo group **(D)**, and the left ventricular ejection fraction (LVEF) in each of the four groups **(E)** and in the combined YS-1402/placebo group **(F)**, measured using cardiac computed tomography. The data are presented for each patient. *p < 0.05 in the YS-1402 group between the preoperative and postoperative (i.e., at 26 weeks) values.

Serial changes in the left ventricular end diastolic volume index (LVEDVI), left ventricular end systolic volume index (LVESVI), and left ventricular ejection fraction (LVEF) measured with CCT are shown in Figure 2. No significant difference existed between the groups in any parameter (Figures 2A, C, E). However, compared to the preoperative values in the YS-1402 group as a whole, each parameter significantly improved 26 weeks postoperatively (LVEDVI: 37 ± 45 mL/m^2^ vs. 115 ± 44 mL/m^2^, *p* = 0.0358; LVESVI: 92.4 ± 44 mL/m^2^ vs. 67.5 ± 39 mL/m^2^, *p* = 0.0012; and LVEF: 35.1% ± 7.1% vs. 44.8% ± 12.7%, *p* < 0.0001) (Figures 2B, D, F). However, the pre- and postoperative values were not significantly different in the placebo group. The same tendency was observed with values measured with UCG (Supplementary Figure 1).

### Increased myocardial blood flow in the YS-1402 group

**FIGURE 3:**
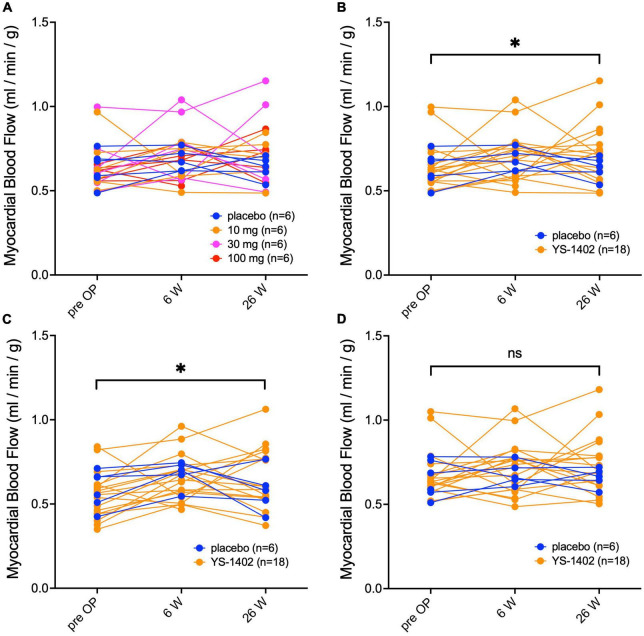
Serial measurements of myocardial blood flow at rest. Serial changes in myocardial blood flow at rest measured with NH_3_-PET for the global region in each of the four groups **(A)**, the combined YS-1402/placebo group **(B)**, the region on which the YS-1402/placebo was placed [**(C)** i.e., YS-1402 site], and the region on which the YS-1402/placebo was not placed [**(D)** i.e., non-YS-1402 site] combined with the YS-1402/placebo group. The data are presented for each patient. *p < 0.05 in the YS-1402 group between the preoperative and postoperative (i.e., at 26 weeks) values.

Serial changes in MBF at rest were assessed with NH_3_-PET. The value of the MBF was obtained as the average for each lesion in the 17-segment model. Based on these values of the 17-segment model, the average values of the global lesion (i.e., the YS-1402 site), which are shown in Table 3, and the non-YS-1402 site were calculated. No significant difference existed between the groups in any of the aforementioned values (Figure 3A). However, compared to the preoperative values in the YS-1402 group as a whole, values of the global and YS-1402 sites had significantly increased at 26 weeks postoperatively (Figures 3B, C) (global: 0.646 ± 0.14 mL/min/g vs. 0.7178 ± 0.18 mL/min/g, *p* = 0.0292; YS-1402 site: 0.554 ± 0.14 vs. 0.650 ± 0.18, *p* = 0.0233; and non-YS-1402 site: 0.654 ± 0.13 vs. 0.729 ± 0.17, *p* = 0.053), although no significant difference existed for the non-YS-1402 site in the YS-1402 groups or for each value in the placebo group (Figure 3D).

### Improved heart failure symptoms after CABG

With regard to heart failure symptoms, most patients had a NYHA II or III classification preoperatively. However, the NYHA classification improved postoperatively in all groups without significant differences between the groups (Supplementary Figure 2). BNP in the serum once increased postoperatively and thereafter serially decreased (Supplementary Figure 3) with no significant differences between the groups. Differences similarly were not significant between the YS-1402 and placebo groups.

## Discussion

In this study, we hypothesized that YS-1402 combined with CABG would improve the myocardial blood flow of patients with ICM to induce functional recovery. In this in-human clinical trial of YS-1402 combined with CABG for ICM, no severe adverse event related to YS-1402 occurred. The pharmacokinetics showed that the C_max_ of ONO-1301 in all patients was less than the NOAEL in the oral administration Phase I study. However, perioperative pleural effusion was significantly increased in the YS-1402 groups. Even though these events could be safely managed using diuretics or drainage, careful management is needed for further clinical applications of this drug.

The therapeutic effects of YS-1402 was not significantly different between the YS-1402 and placebo groups for LVEF, MBF, BNP, and heart failure symptoms postoperatively. This trial did not demonstrate significant positive effects of YS-1402 administered on the LV of ICM hearts, compared to the hearts that underwent the placebo operations. However, the MBF in the YS-1402 groups had significantly increased at 26 weeks postoperatively, although no significant difference between pre- and postoperative MBF values existed in the placebo group. Investigators have reported that MBF does not increase after CABG at rest (9), suggesting that, even if blood flow is remedied by CABG by grafting to the central side of the coronary artery, blood flow in the myocardial tissue does not sufficiently improve in patients with ICM because of the disrupted peripheral arterial network in the myocardium. Nevertheless, MBF at rest 26 weeks postoperatively was significantly increased in the YS-1402 groups, especially in the YS-1402 site, which suggested that the microvascular network was reconstructed in the myocardium by the local administration of YS-1402. Further studies should be conducted to confirm this finding and the therapeutic efficacy of YS-1402 for patients with ICM.

ONO-1301 was originally developed as an antiplatelet aggregating drug (5); however, it was discontinued because gastrointestinal symptoms developed at blood concentrations representing its effectiveness in the Phase I clinical trial. Previous preclinical studies (7, 8, 10–14) have shown that the local administration of ONO-1301 enhanced the cytokine paracrine effect, thereby leading to its development as an anti-heart failure drug (i.e., a drug-repositioning drug). To date, a possible therapeutic mechanism of ONO-1301 for ICM is its angiogenesis effect, which might have had a role in the increase in MBF in the YS-1402 groups. ONO-1301 was proven to directly activate endothelial cells and vascular smooth muscle cells through the IP receptor to induce the release of protective factors such as hepatocyte growth factor, VEGF, or SDF-1, into the damaged cardiac tissue to reconstruct vessels (15). In addition, hepatocyte growth factor, which was induced by ONO-1301, could inhibit TGF-β-mediated fibrosis (14). Cardiac fibrosis is a crucial mechanism in the regulation of cardiac function via LV remodeling. In this study, in the YS-1402 groups, a significant reduction in left ventricular volume and an increase in LVEF were observed, although no significant improvements existed in the placebo group. This finding may suggest that left ventricular remodeling was suppressed by the fibrosis-suppressing effect of YS-1402.

In this study, YS-1402 was synthesized as a sustained-release preparation using poly(ethylene adipate-co-D,L-lactic acid) infiltrated into a gelatin sheet and attached to the surface of the heart to suppress the systemic adverse effects of ONO-1301 and maximize the local therapeutic effects. Thus, the blood concentration of ONO-1301 gradually increased after its administration, depending on the dose of YS-1402, peaked in approximately 2 weeks, and disappeared after 8 weeks, which confirmed the sustained release of ONO-1301. Moreover, considering that the C_max_ at the maximum NOAEL was 23.69 ng/mL in the Phase I oral administration study of ONO-1301, the observation that the peak blood concentration of ONO-1301 at the maximum dose of YS-1402 (100 mg) in this study was approximately 10 ng/mL suggested that 100 mg of YS-1402 is a dose that is safe. No significant differences existed in the occurrence of severe adverse effects and evaluation of therapeutic effects between the 10-, 30-, and 100-mg YS-1402 groups; however, the blood concentration of ONO-1301 was sufficiently safe, even at the maximum dose of 100 mg. Therefore, a dose of 100 mg or more should be investigated in further clinical trials. In addition, it may be necessary to consider the other local delivery methods of this drug such as intracardiac injection to avoid the side effects of pleural effusion.

The limitations of this study include the small number of patients, which may not have allowed the proper evaluation of the therapeutic efficacy of YS-1402. In particular, the viability of the myocardium in ICM affects the therapeutic effect of YS-1402, as well as the therapeutic effect of CABG. In this study, the viability of the myocardium in patients with ICM may be biased in a small number of patients in each group, which may affect the evaluation of the therapeutic effect. After confirming the safety assessment of YS-1402 administration on the heart in this clinical trial, a further Phase IIb/III clinical trial with a larger number of patients should be considered to reconfirm the therapeutic efficacy of YS-1402.

The local administration of YS-1402 on ICM hearts was safely and tolerably performed as a concomitant surgery of CABG. Further studies should be considered to investigate the therapeutic efficacy of YS-1402 for ICM.

## Materials and methods

### Study design

**FIGURE 4:**
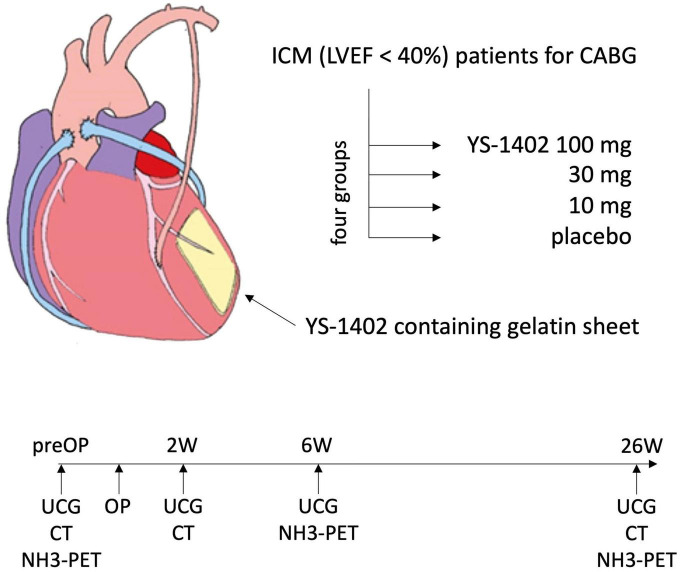
Study scheme. ICM, ischemic cardiomyopathy; LVEF, left ventricular ejection fraction; UCG, ultrasonic cardiogram.

The local administration of YS-1402 on the heart requires open heart surgery. To minimize invasiveness, we planned it as concomitant surgery with CABG. The dose of YS-1402 used was 10–100 mg, based on doses reported in previous preclinical studies (6). Patients with ICM who were included in this study, based on the following inclusion/exclusion criteria, were assigned in a double-blinded manner to four groups: placebo, 10-mg YS-1402, 30-mg YS-1402, and 100-mg YS-1402. In addition to observing adverse events, serial changes in cardiac function, assessed with echocardiography (i.e., ultrasonic cardiogram [UCG]) or electrocardiogram-gated cardiac computed tomography (CCT), and serial changes in myocardial blood flow (f), assessed with (13) N-ammonia positron emission tomography (NH_3_-PET), were recorded for 6 months postoperatively (Figure 4).

### Inclusion criteria

Patients who met all of the following criteria were included: (1) diagnosed with ischemic cardiomyopathy and undergoing coronary artery bypass surgery by thoracotomy; (2) a left ventricular ejection fraction ≤ 40%, based on echocardiography within 4 weeks before consent for participation in the study or 4 weeks preoperatively; (3) experiencing congestive heart failure, even when treated with maximum medication therapy such as digitalis, diuretics, angiotensin converting enzyme inhibitors, angiotensin II receptor blockers, and beta blockers; and (4) age of 20–80 years at the time of consent for participation in the study.

### Exclusion criteria

Patients who met any of the following criteria were excluded: (1) severe organic valvular disease or cardiovascular abnormalities, including an aortic aneurysm, that are deemed by the investigator to affect any procedure of the clinical trial; (2) irreversible organ failure other than the heart; (3) malignant tumors; (4) suspected or confirmed diabetic proliferative retinopathy; (5) confirmed or possible pregnancy, breastfeeding women, men, and fertile patients who do not agree to use appropriate contraceptive methods during the study period; (6) refusal of blood transfusion; (7) renal dysfunction (chronic kidney disease [CKD] of stage D or worse) and hepatic dysfunction (Child-Pugh B or C); (8) history of alcoholism or drug addiction within 6 months before registration; (9) severe psychosis or psychiatric symptoms deemed by a doctor to make participating in this clinical trial difficult for the corresponding patient; (10) gelatin hypersensitivity; (11) aprotinin hypersensitivity; (12) treatment with coagulation promoter (e.g., snake venom preparation), antifibrinolytic agent, and aprotinin preparation; (13) patients within 6 months after the end of other clinical trials; and (14) patients who were deemed inappropriate for this study by an investigator.

### Operative procedure

Coronary artery bypass grafting and transplantation of YS-1402 were performed through a median sternotomy. The detailed CABG procedure, including target vessels, graft design, and need of cardiopulmonary bypass or intra-aortic balloon pumping, was determined preoperatively, after a discussion between the heart team using the same indications as those for a normal CABG. YS-1402 transplantation was performed after the CABG procedure and just before closing the chest. YS-1402 was added to two gelatin sheets (20 mm × 60 mm), which were subsequently placed on the left ventricle (LV) at the ischemic site. The ischemic site of the LV was determined, based on preoperative NH_3_-PET findings and the operative observation of the surface of LV. The YS-1402 sites were recorded, based on the LV 17-segment model number.

### Data collection

The preoperative characteristics of the patients were recorded during the screening periods of 4 weeks. In addition to collecting adverse events postoperatively, serial changes in cardiac function were assessed using UCG preoperatively and at 2, 6, and 26 weeks postoperatively, and assessed using CCT preoperatively and at 2 and 26 weeks postoperatively. Furthermore, serial changes in myocardial blood flow (MBF) during rest were measured preoperatively using NH_3_-PET and at 6 and 26 weeks postoperatively (Figure 4).

To assess symptoms of heart failure, we collected subjective symptoms, based on the New York Heart Association (NYHA) Functional Classification, and measured the serum concentration of brain natriuretic peptide (BNP). All data were collected by independent clinical research coordinators and subsequently analyzed in a central data center for this clinical trial.

### Statistical analysis

The baseline characteristics, operative procedure, and observed adverse events were tabulated using standard descriptors of central tendency and variability, including the median (interquartile range). Differences between groups were analyzed using parametric and non-parametric tests, as appropriate.

If the data used for analysis were missing, values were treated as missing, and no special complement processing was performed statistically. All analyses were conducted using JMP Pro version 14 (SAS Institute Inc., Cary, NC, USA) and GraphPad Prism version 9 (GraphPad Software Inc., San Diego, CA). A value of *p* < 0.05 was significant.

## Data availability statement

The original contributions presented in this study are included in this article/Supplementary material, further inquiries can be directed to the corresponding author.

## Ethics statement

The studies involving human participants were reviewed and approved by the Ethical Review Committee of the Osaka University Hospital (Suita, Japan). The patients/participants provided their written informed consent to participate in this study.

## Author contributions

TK, DY, MK, RM, AK, and YM conducted the surgeries. YS and SM designed the clinical trial. TK and TM wrote the manuscript. All authors contributed to the article and approved the submitted version.

## Supplementary material

Supplementary Figure 1 Serial echocardiographic assessments of the left ventricular ejection fraction. Serial changes in the left ventricular ejection fraction (LVEF) in each of the four groups **(a)** and in the combined YS-1402/placebo group **(b)**. The data are presented as the mean ± the standard error. **p* < 0.05 in the YS-1402 group between the preoperative and postoperative (i.e., at 6 or 26 weeks) values.

Supplementary Figure 2 Serial assessment of heart failure symptoms. Serial changes in heart failure symptoms, assessed using the NYHA classification in each of the four groups. NYHA, New York Heart Association.

Supplementary Figure 3 Serial measurements of the serum brain natriuretic peptide concentration. Serial changes in the serum concentration of brain natriuretic peptide in each of the four groups **(a)** and in the combined YS-1402/placebo group **(b)**. The data are presented as the mean ± the standard error.

Supplementary Figure 4 Serial measurements of the serum AST, ALT, and Creatine Kinase concentration. Serial changes in the serum concentration of aspartate transaminase (AST), alanine transaminase (ALT), and Creatine Kinase in each of the four groups **(A,C,E)** and in the combined YS-1402/placebo group **(B,D,F)**. The data are presented as the mean ± the standard error.
