# Supplementary figures and images for: Enhanced myocardial blood flow in ischemic cardiomyopathy by a slow-release synthetic prostacyclin agonist combined with coronary artery bypass grafting: The first human study in a Phase I/IIa clinical trial
Source: Front Cardiovasc Med. 2023 Jan 25;10:1047666. doi: 10.3389/fcvm.2023.1047666 (PMC9905424; doi:10.3389/fcvm.2023.1047666)

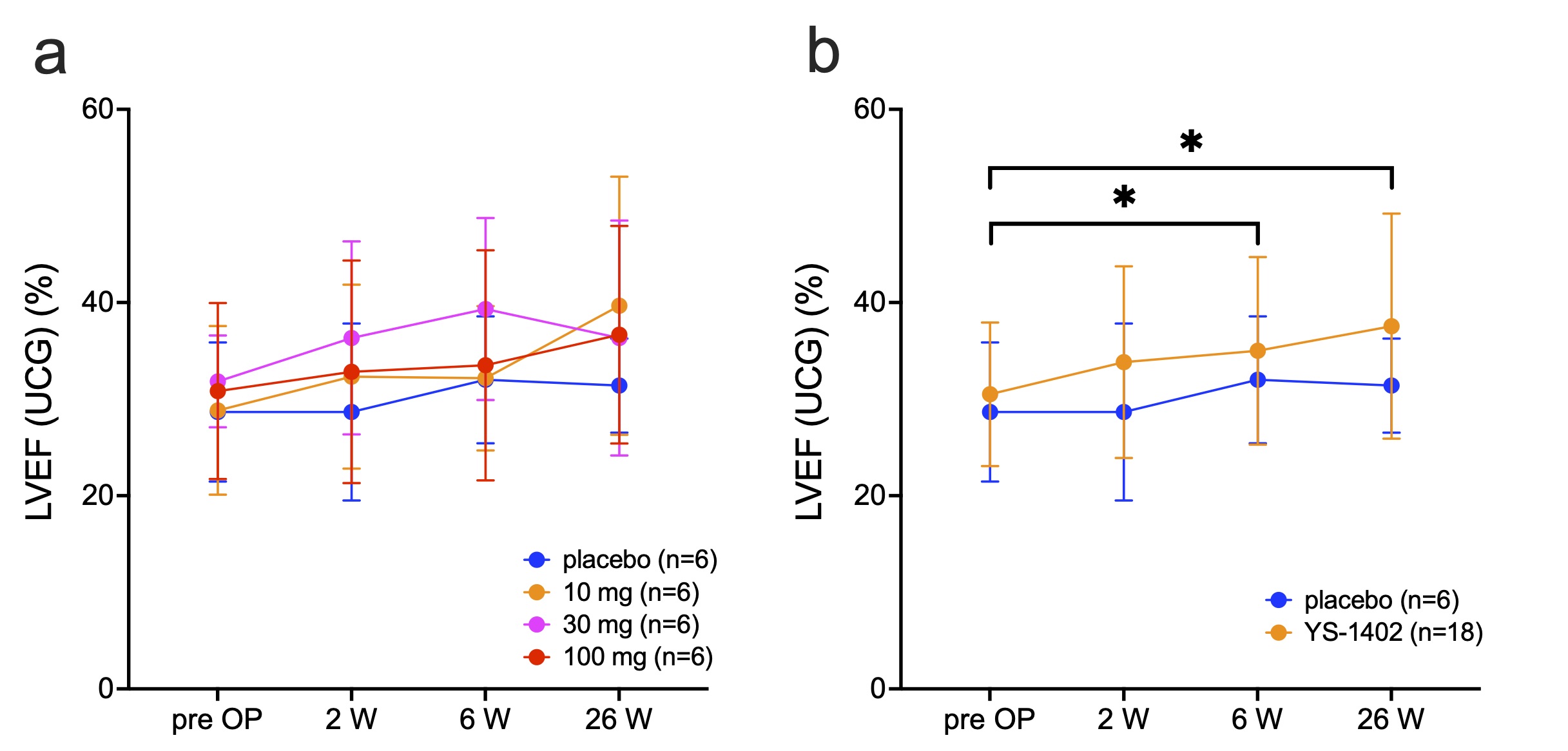

Supplement: Supplementary Figure 1 — Serial echocardiographic assessments of the left ventricular ejection fraction. Serial changes in the left ventricular ejection fraction (LVEF) in each of the four groups (a) and in the combined YS-1402/placebo group (b). The data are presented as the mean ± the standard error. *p < 0.05 in the YS-1402 group between the preoperative and postoperative (i.e., at 6 or 26 weeks) values. [file Image_1.JPEG]

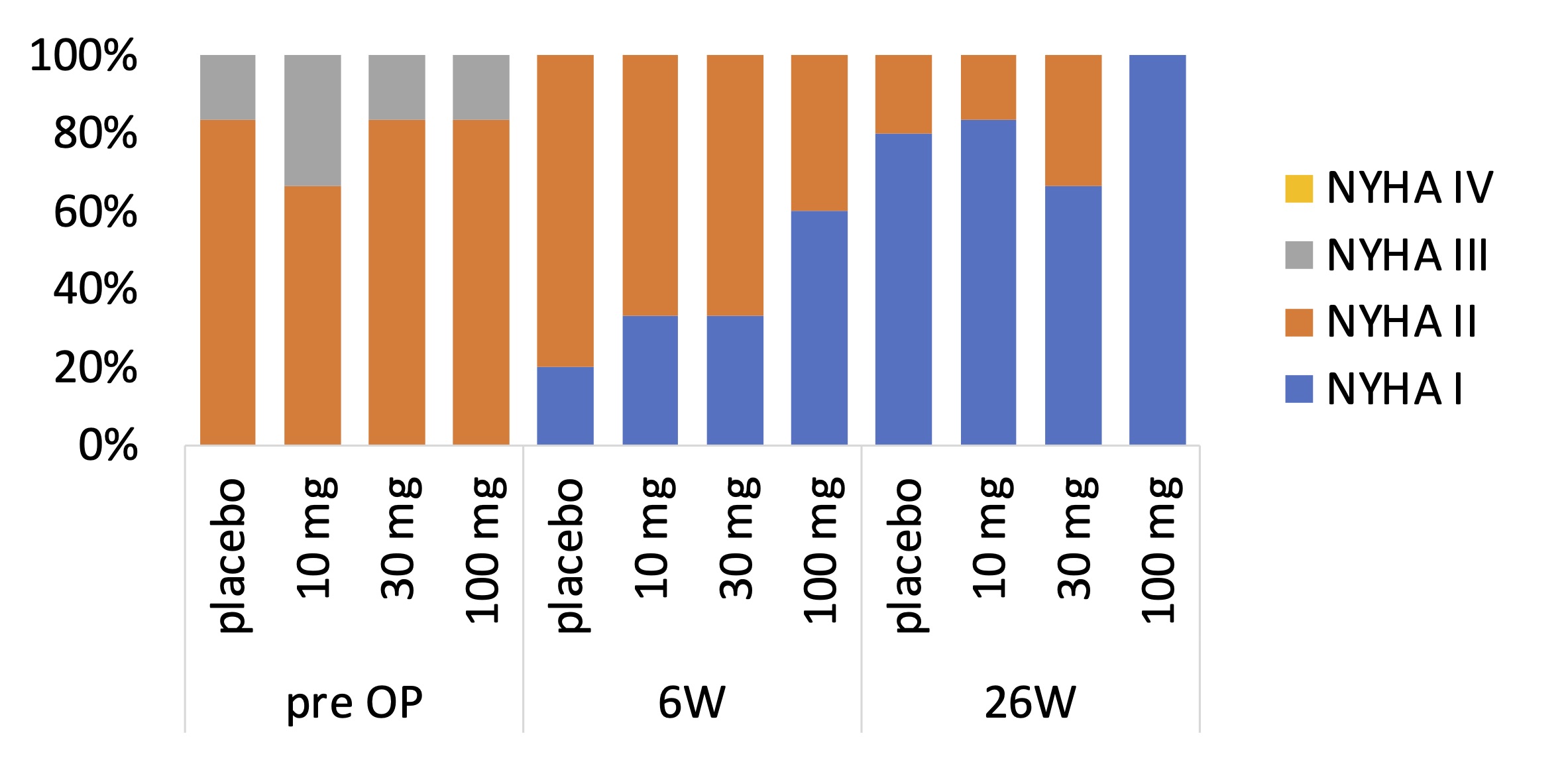

Supplement: Supplementary Figure 2 — Serial assessment of heart failure symptoms. Serial changes in heart failure symptoms, assessed using the NYHA classification in each of the four groups. NYHA, New York Heart Association. [file Image_2.JPEG]

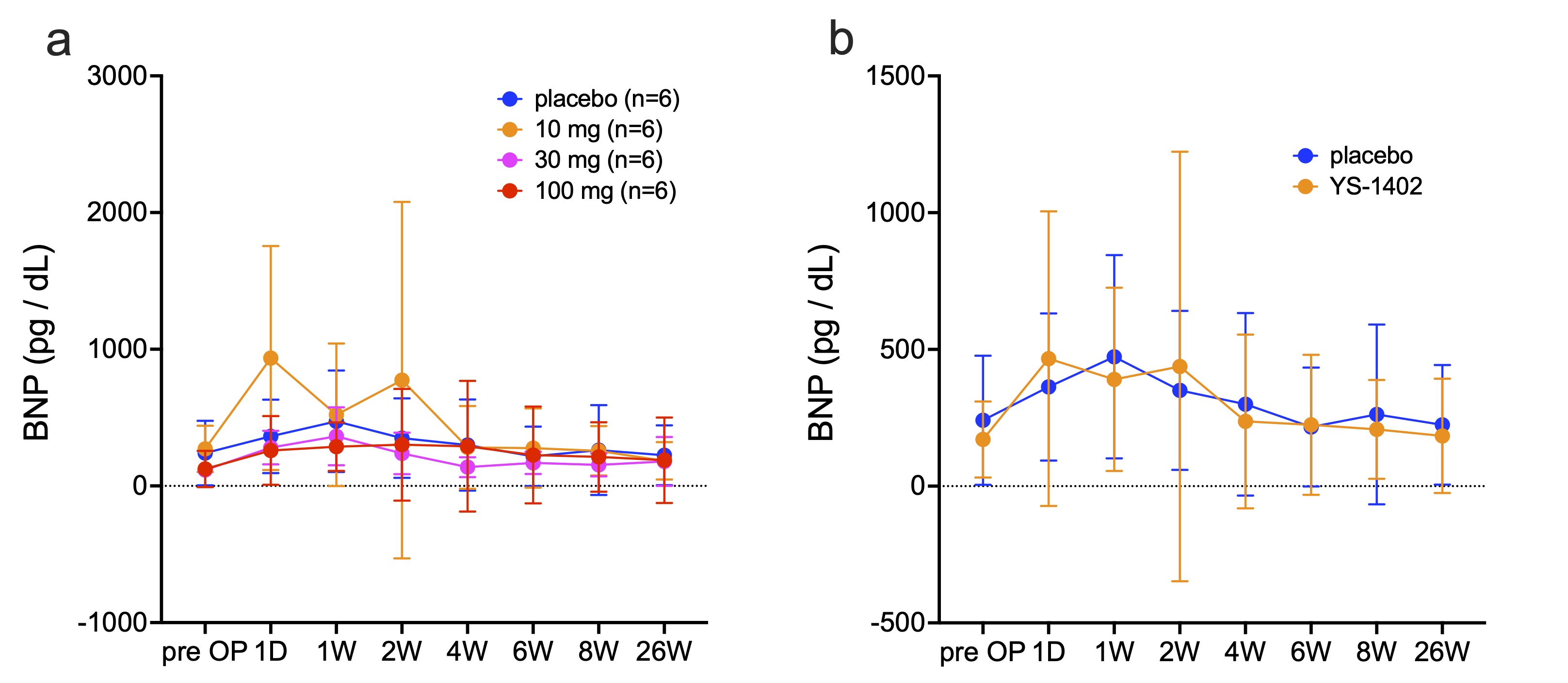

Supplement: Supplementary Figure 3 — Serial measurements of the serum brain natriuretic peptide concentration. Serial changes in the serum concentration of brain natriuretic peptide in each of the four groups (a) and in the combined YS-1402/placebo group (b). The data are presented as the mean ± the standard error. [file Image_3.JPEG]

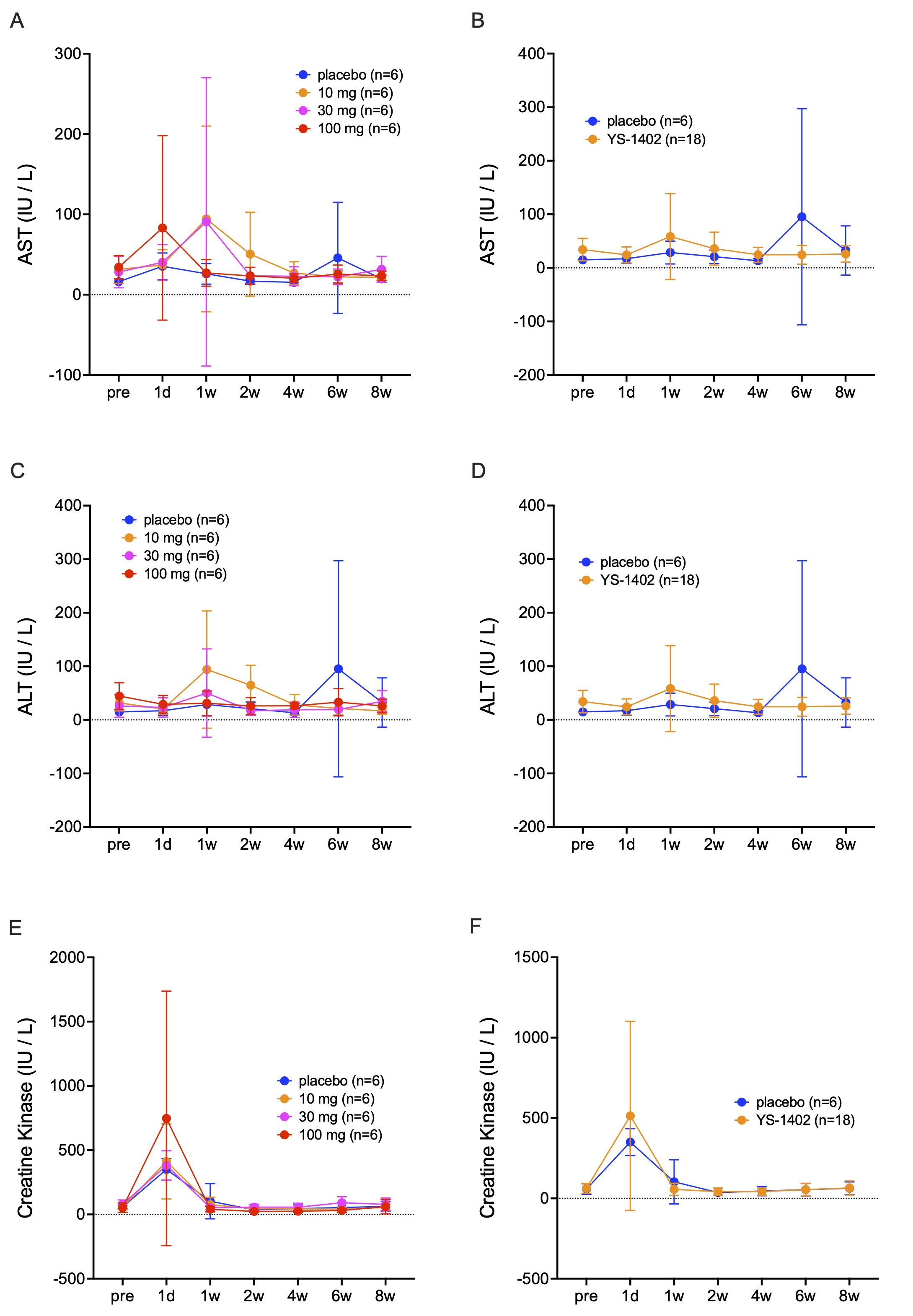

Supplement: Supplementary Figure 4 — Serial measurements of the serum AST, ALT, and Creatine Kinase concentration. Serial changes in the serum concentration of aspartate transaminase (AST), alanine transaminase (ALT), and Creatine Kinase in each of the four groups (A,C,E) and in the combined YS-1402/placebo group (B,D,F). The data are presented as the mean ± the standard error. [file Image_4.JPEG]
